# A scoping review of the literature on interventions to reduce stress and burnout among teachers.

**DOI:** 10.1192/j.eurpsy.2024.444

**Published:** 2024-08-27

**Authors:** A. Belinda, Y. Wei

**Affiliations:** Department of psychiatry, University of Alberta, Edmonton, Canada

## Abstract

**Introduction:**

Experience of chronic stress among professionals is a risk factor for poor mental and physical well-being. There is limited knowledge regarding the kinds of interventions, and outcomes achieved using different modalities to address stress and burnout among teachers.

**Objectives:**

To undertake a scoping review of recent literature to determine psychological interventions and reported outcomes related to stress and burnout among teachers.

**Methods:**

The PRISMA-ScR (Preferred Reporting Items for Systematic Reviews and Meta-Analyses extension for Scoping Reviews was followed. Relevant search terms were used to determine different interventions adopted to reduce teachers’ stress and burnout. Articles published between 2018 and 2022 were identified using five bibliographic databases. Relevant articles were extracted, reviewed, collated, and thematically analyzed, and findings s were summarized.

**Results:**

Forty studies conducted in Asia, North America, Oceania, Europe, and Africa, met the inclusion criteria. Sixteen kinds of burnout and stress-reduction interventions were identified. The most popularly studied intervention were Mindfulness-Based Interventions alone or in combination with yoga or Cognitive Behavioural Therapy (CBT), followed by Rational Emotive Behavioral Therapy (REBT). Mindfulness-Based Interventions led to decreased overall Teacher Stress Inventory (TSI) and emotional exhaustion subscale scores. REBT, primarily used with special education teachers, especially in Africa, has also shown positive results. Other interventions reporting positive outcomes include Inquiry-Based Stress Reduction (IBSR), the Stress Management and Resiliency Training Program (SMART), Cyclic Meditation, Group Sandplay, Progressive Muscle Relaxation, Autogenic Training, Sport-Based Physical Activity, Emotional Intelligence Ability Models and Christian Prayer and Prayer-Reflection

**Image:**

**Figure:**
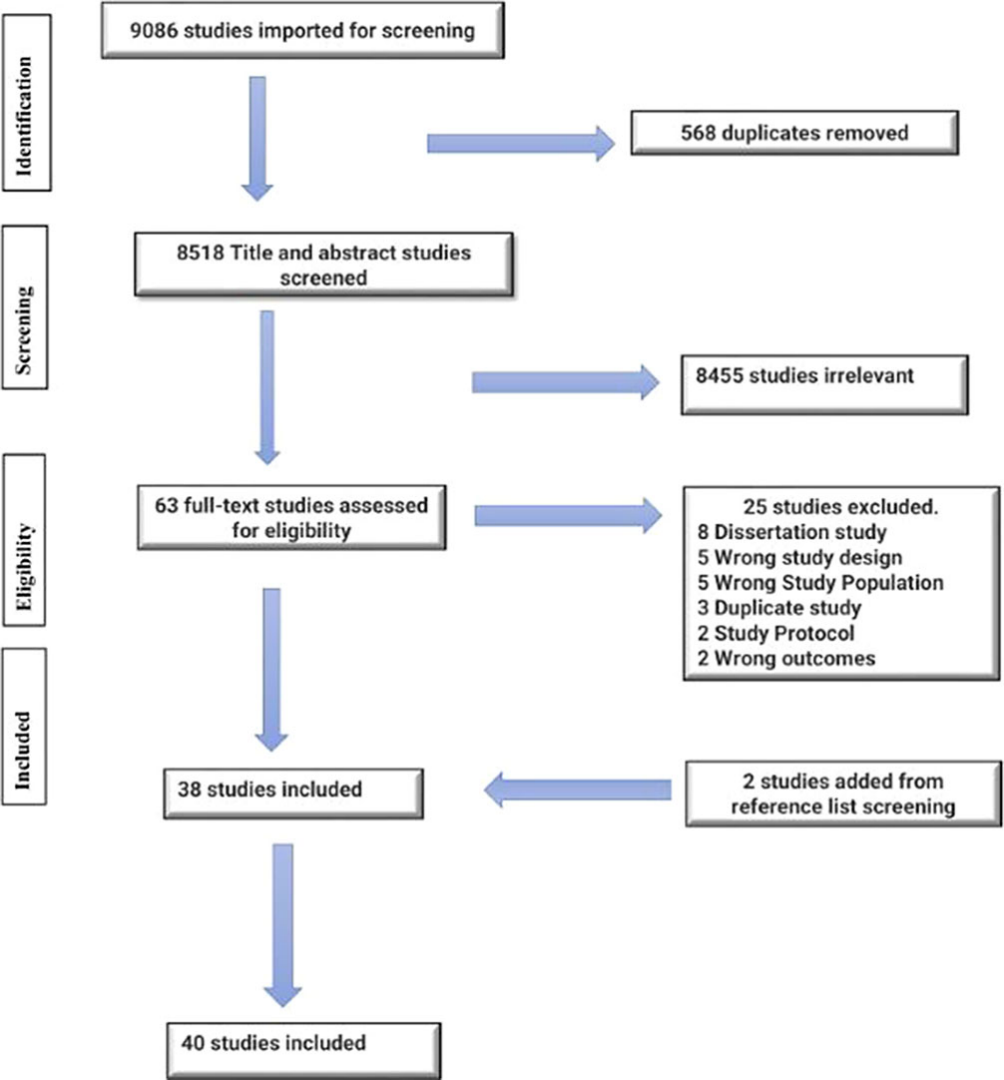

**Conclusions:**

Stress and burnout can have a negative impact on teachers and, very often, on the students they teach. Implementing suitable school-based interventions are necessary to improve teachers’ stress-coping ability, reduce the likelihood of burnout and improve general well-being. Policymakers, governments, school boards and administrators should prioritize the implementation of school-based awareness and intervention programs to mitigate teacher stress and burnout.

**Disclosure of Interest:**

None Declared

